# Increasing Cation Ion Symmetry Reduces Ionic Liquid Ordering in Thin Films

**DOI:** 10.1021/acs.jpcb.4c04413

**Published:** 2024-11-05

**Authors:** Michael Blake Van Den Top, Andrew Horvath, Spyridon Koutsoukos, Frederik Philippi, Daniel Rauber, Tom Welton, Scott K. Shaw

**Affiliations:** † Department of Chemistry, University of Iowa, Iowa, Iowa 52242, United States; ‡ Department of Chemistry, Imperial College, London SW7 2AZ, U.K.; § Department of Chemistry, Saarland University, Campus B 2.2, 66123 Saarbrücken, Germany

## Abstract

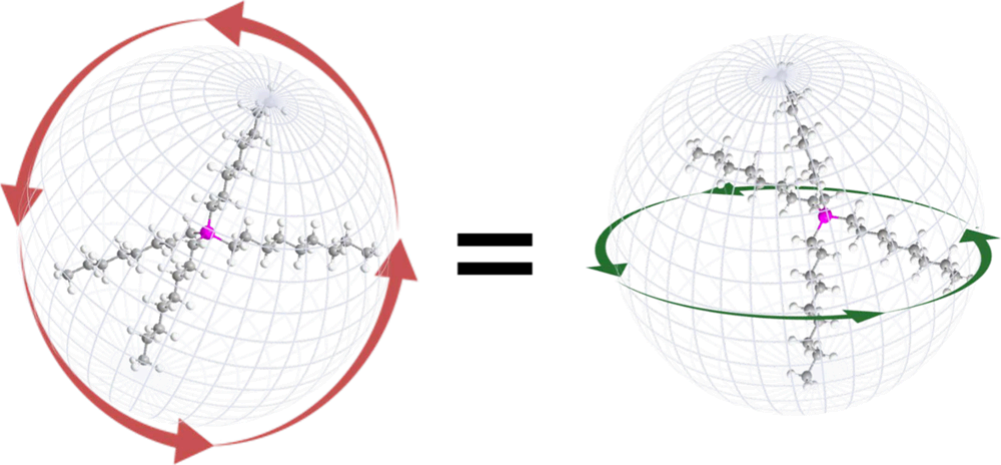

Ionic liquids have been shown to form extended ordered structures near surfaces and in bulk. Identifying fundamental driving force(s) for this organization has been elusive. In this paper, we test a hypothesis that the ionic liquid asymmetry, inherent in many of the IL formulations to frustrate crystallization, is a significant contributor to the observed ordering. We have carried out measurements to track the ordering of ionic liquids composed of “spherical” cations, namely, tetraoctylphosphonium ([P8888]) and tetra(propoxymethyl)phosphonium [P(3O1)4] paired with tetracyanoborate anion [B(CN)4]. Analysis of the infrared signatures for films of these ionic liquids shows very little evidence of ordered structures. These liquids instead remain in a more isotropic environment even when confined to volumes of few micrometer dimensions.

## Introduction

Ionic liquids (ILs) are salts that are molten at temperatures of interest. The term is used to infer that these salts are melting at lower temperatures than traditional salts such as sodium chloride. ILs are comprised of bulky and often organic, asymmetric ions.^1,2^ The resulting steric bulk and charge delocalization lowers melting points (from ca. 800 °C for NaCl) to around or even below room temperature for many ionic liquids. Further, ILs offer several potentially useful properties such as high thermal stability, negligible vapor pressure, and tunable viscosity.^3,4^ ILs with ether functional groups have attracted attention due to lower viscosities compared to fully alkylated tails in ILs of otherwise similar mass and structure.^5,6^

ILs have become attractive for use in many fields, including energy storage and lubrication in extreme environments.^2−4^ Many current and potential IL applications involve the unusual behavior of IL materials at solid- or vapor-phase interfaces. It is well-known that the interfacial phase can exhibit very different behaviors from the bulk.^7−11^ These differences arise from fluid molecules responding to the adjacent interface to spontaneously form ordered structures. These structures result in familiar effects such as surface tension, and they generally decay over distances of several molecular diameters (a few nanometers) from the interfacial plane. However, these ordered structures of ionic liquids have been reported to extend from several nanometers to micrometer distances,^12,13^ and even persist in the bulk phase,^14^ marking a significant departure from traditional understanding of chemical interfaces.

Exploring these unique behaviors has been an exciting area for chemistry, resulting in several significant advances. For example, it has been shown that many ILs display a sponge-like structure consisting of a charge network composed of the polar charged head groups of the IL cations and ions with the interstitial region being comprised of the apolar tails of the ions.^5,15−17^ These results show that the time scales over which these structures form and evolve differ between the polar and apolar regions of the fluid structure. These different time scales imply the presence of two distinct yet linked chemical environments present within ILs.

The Shaw group has previously reported IL ordered structures that require time scales of minutes to hours to form ordered interfacial structures that extend over several micrometers. ILs’ slow dynamics have been reported previously and are generally exhibited as high viscosity in experimental measurements.^18^ Many ILs have room temperature viscosities over 100 cP, whereas the viscosity of water is ca. 1 cP at room temperature.

The process by which the fluid film reorganizes to adopt an ordered structure is called maturation, but the root driving force for this process has remained an ongoing area of investigation. Previous work by the Shaw group probed how bulk viscosity affects the time scale over which IL films “mature” into ordered structures.^19^ A number of bis(trifluoromethylsulfonyl)imide ([TFSI]^−^)-based ILs were examined across viscosity variation and results showed a direct correlation between the viscosity of the IL and the time to maturation.^19,20^ We have since tested ILs with more symmetrical anions, e.g., the [B(CN)_4_]^−^ anion which shows significantly different maturation behaviors, supporting a hypothesis that ion (a)symmetry creates a driving force to induce order within the film. Leading theories to attribute these behaviors to specific conditions of the experiment or general properties of ionic liquids have included that the maturation process was driven by shear flow, as in liquid crystals, or that the ionic liquid ordered structure was “templated” by the adjacent solid substrate surface and slowly extended into the liquid phase away from the surface. Experiments with different substrate surface chemistries, and other measurements that show similar ordering behaviors without any shear flow have made these theories unlikely. One remaining possibility is that the inherent asymmetry of the IL ions is driving the ordering transition, despite their relatively small size compared to traditional liquid crystals. This paper examines the likelihood of this theory directly by using quasi-spherical anions and cations to create an ionic liquid, and examining this liquid for any of the ordering or maturation effects described in the prior publications mentioned above.

The current work extends our studies of ion symmetry to include quasi-spherical cations, namely tetraoctylphosphonium ([P8888]^+^) and tetrakis(3-methoxypropyl)phosphonium [P(3O1)_4_]^+^ paired with the also spherical tetracyanoborate anion [B(CN)_4_].^21^ With this work we aim to determine if these quasi-spherical ions, which have significantly less asymmetry that those in our prior works, will diminish the extent to which the thin IL films form ordered structures supporting the theory that the ion symmetry is the driving force for maturation. Specifically, we predict that films comprised of these symmetric ILs are much less likely to form any particular ordered structure resulting in a more isotropic film environment. The -alkyl and -ether functionalized phosphonium cations here provide a significant chemical space for testing this hypothesis.

## Experimental Section

### Materials

The ILs tetraoctylphosphonium tetracyanoborate ([P8888][B(CN)_4_]) and tetrakis(3-methoxypropyl)phosphonium tetracyanoborate ([P(3O1)_4_][B(CN)_4_]) were synthesized as described previously^21^ and dried under reduced pressure for >5 days to remove residual water and volatile impurities. All ILs are stored under nitrogen atmosphere when not in use. Water content is monitored before each experiment via Karl Fischer titration (see below).

Infrared spectroscopic measurements on IL films are acquired using polycrystalline silver disks as solid substrates to support the IL films. These disks are cut from 99.999% purity silver rod (ESPI metals, Portland, OR) and polished to a mirror finish using progressively finer grit polishing pads (starting with 600 then 1000 grit sandpapers followed by 9.5, 3.0, 1.0, and 0.3 μm aluminum oxide powder on Buehler polishing pads) followed by a chemical polish using chromic acid. The Ag surface cleanliness and optical constants are measured by atomic force microscopy (AFM) and spectroscopic ellipsometry, respectively. The RMS roughness is better than 3 nm and the *n* and *k* values determined by ellipsometry match those reported for bare metals.^22^

### Instrumental Methods

#### Karl Fischer Titration

Water content was determined using a Metrohm 831 Karl Fischer (KF) titrator with a two-reagent diaphragm cell. The outer cell consisted of Hydranal Coulomat AG anolyte (Fluka Analytical) or Aqualine Electrolyte AG (Fisher Chemical) and the inner cell of Hydranal Coulomat CG catholyte (Fluka Analytical). Hydranal Water Standard 1.0 (Fluka Analytical) was used to calibrate the instrument after changing the Karl Fischer reagents and periodically during these experiments. The sample was stirred vigorously before every Karl Fischer measurement to ensure homogeneity.

#### Dynamic Wetting

Wetting experiments to create the thin films were performed in a custom, airtight PTFE cell and motor assembly described previously.^23^ Briefly, substrate surfaces were held in the vertical orientation and rotated at controlled velocities through a fluid droplet dispensed by a capillary near the bottom of the Ag substrate, which causes a film to be extruded onto the solid. The film was probed near the apex of the rotation with various spectroscopic techniques, e.g., spectroscopic ellipsometry and infrared-reflection–absorption spectroscopy (IRRAS). The interior of the cell was purged with dry N_2_ gas (UHP 99.999%, Praxair) to remove water, CO_2_, and oxygen.

#### FTIR

A Thermo-Nicolet iS50 Fourier transform spectrometer with liquid N_2_ cooled MCT-A detector was used to acquire FTIR spectra. For transmission measurements, a ca. 10 μL aliquot of the sample was pressed between two CaF_2_ plates and spectra were averaged over 128 scans (2 min). Duplicate spectra were obtained for each sample. The CaF_2_ plates were cleaned using copious acetone rinses followed by drying under a dry nitrogen stream in between measurements.

#### IRRAS

Spectroscopic characterization of the films was performed in a reflection geometry using infrared reflection absorption spectroscopy (IRRAS). IRRAS spectra were acquired using the same Thermo-Nicolet iS50 FTIR spectrometer coupled to an external optical bench with MCT-A detector to accommodate the dynamic wetting cell. The external bench passed the infrared light through a wire grid polarizer to create a p-polarized incident beam. This beam was gently focused onto the sample and the reflected beam was focused onto the MCT-A detector. All spectra were collected at 4 cm^–1^ resolution and averaged over 1000 scans. Background spectra were collected from clean and dry Ag substrates before introducing the fluid film.

#### Ellipsometry

An M-2000 spectroscopic ellipsometer (J.A. Wollam Co., Inc.) was used to measure film thickness via reflection measurements in a cell and sampling geometry similar to that described above. The ellipsometer reports ψ and Δ values as a function of wavelength from 350 to 1000 nm. These values contain information to calculate refractive index, extinction coefficient, and thickness of the film. ψ and Δ values obtained at different rotational velocities were fit to report the film’s thickness. The fitting model consists of a bare silver substrate layer, an intermixed layer, and a general oscillator layer that accounts for small optical absorption by the thickest films.

### Theoretical Methods

Ab initio simulation geometry optimization and calculation of IR spectra were performed at the B3LYP-GD3BJ/6-311+G(d,p) level of theory using the Gaussian software package, revision E.01 as described previously.^21,24^ Three cation geometries were considered. For each cation geometry, the four side chains were prepared in the same conformation, corresponding to either GS1, GS2, or GS3 in reference.^21^ Frequencies were scaled with 0.97. Additional optimizations and frequency calculations were performed in the presence of a solvent, specifically the [BMIM][TFSI] SMD continuum solvent model as a generic IL background.^25^

## Results and Discussion

**Figure 1:**
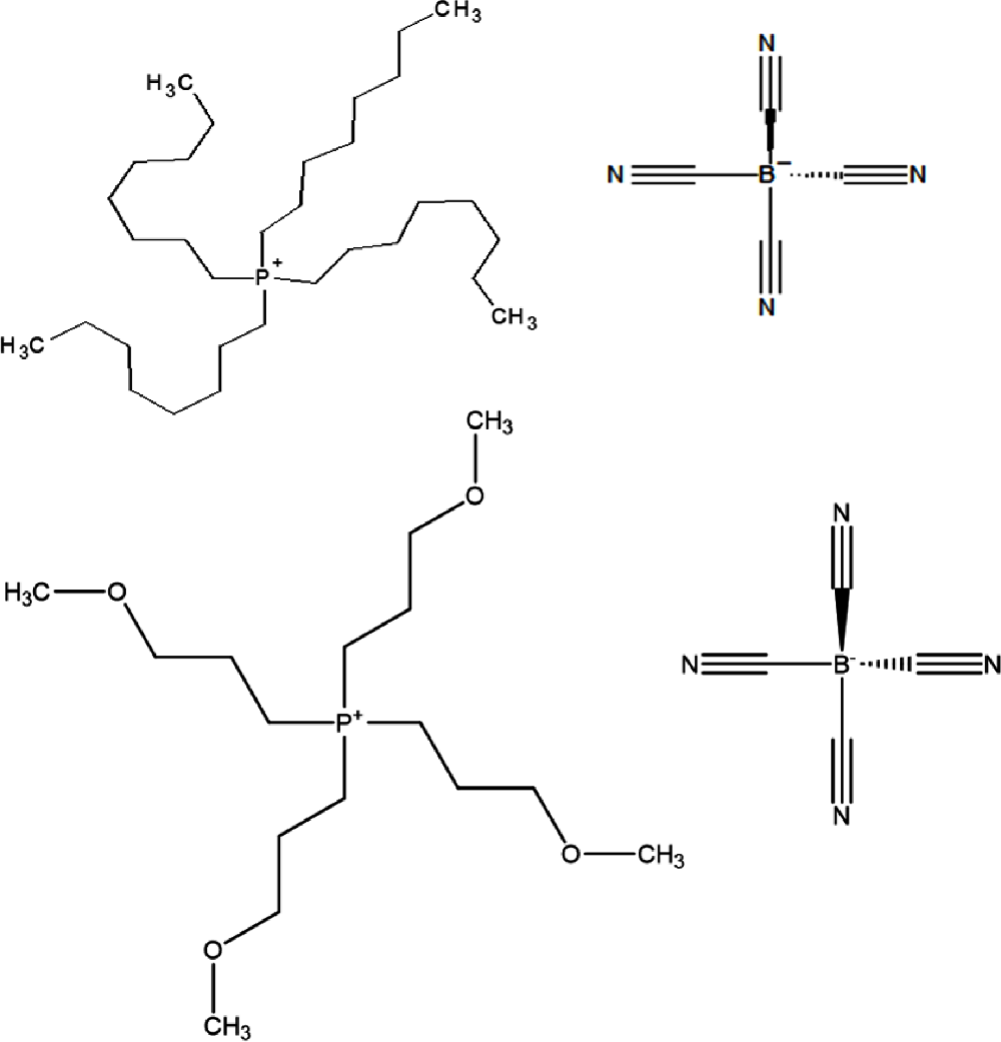
Chemical structures of tetraoctylphosphonium tetracyanoborate ([P8888][B(CN)_4_]) (top) and tetra(propoxymethyl)phosphonium tetracyanoborate ([P(3O1)_4_][B(CN)_4_]) (bottom), the two ILs used in this study.

Two aprotic ILs containing the tetracyanoborate anion: tetraoctylphosphonium tetracyanoborate ([P8888][B(CN)_4_]) and tetra(propoxymethyl)phosphonium tetracyanoborate ([P(3O1)_4_][B(CN)_4_]) were used to study the effects of differing cation symmetry on interfacial film behavior. The molecular structures of both ILs used in this study can be seen in Figure 1. The primary difference between the two ILs is an ether functional group included in the [P(3O1)_4_]^+^ tail.

**Figure 2:**
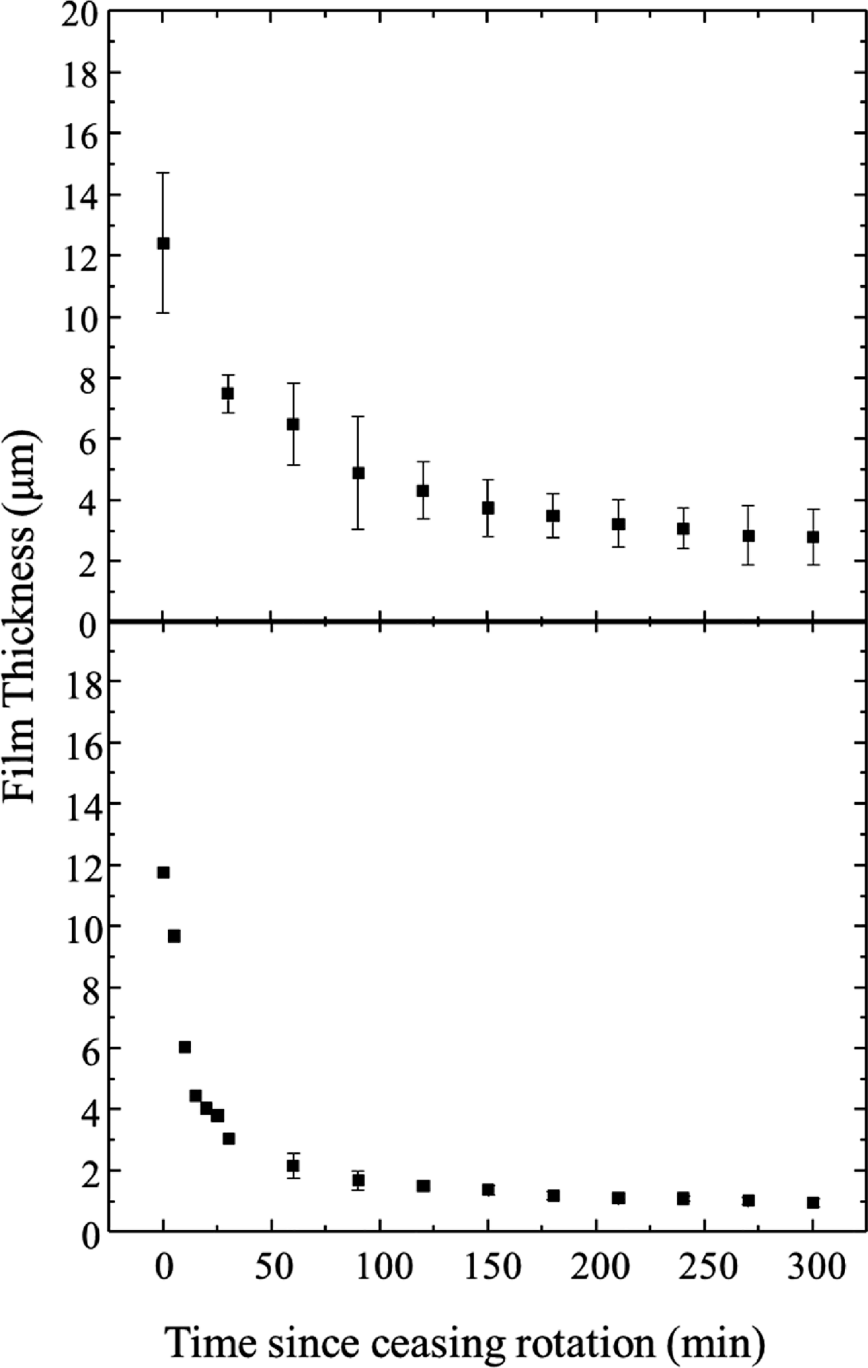
Film thicknesses measured by ellipsometry acquired on a [P8888][B(CN)_4_] film (top) and a [P3O1][B(CN)_4_] film (bottom). Data is collected with the films are rotating and as a function of time after substrate rotation is stopped. Data points represent *n* ≥ 3 trials. Error bars represented standard deviation in the measurement.

Figure 2 shows film thicknesses obtained from ellipsometry measurements of both ILs used in this study. According to the Landau-Levich model, differences in bulk fluid properties (viscosity, density, surface tension) govern the thickness of a fluid film on a solid substrate as it is withdrawn from a bath of the liquid.^26^ To improve comparability between the two ILs being tested here, we designed our experiments such that both ILs start at similar thicknesses at time zero in Figure 2. After the IL film was established on the Ag substrate, the motion of the substrate was stopped. We used ellipsometry to track the film thickness over time. Thickness data were collected every 30 min over a period of 5 h (with the exception of [P(3O1)_4_][B(CN)_4_]’s first 30 min which were collected every 5 min). Both films showed a rapid initial drop in film thickness, followed by asymptotic decays to ca. 3 and 1 m for the tetraalkyl and the ether phosphonium cation, respectively. Error bars in these measurements are standard deviations of *n* > 3 independent sample data sets. The respective thinning behavior is expected based on the bulk viscosities of these liquids, as noted above.

**Figure 3:**
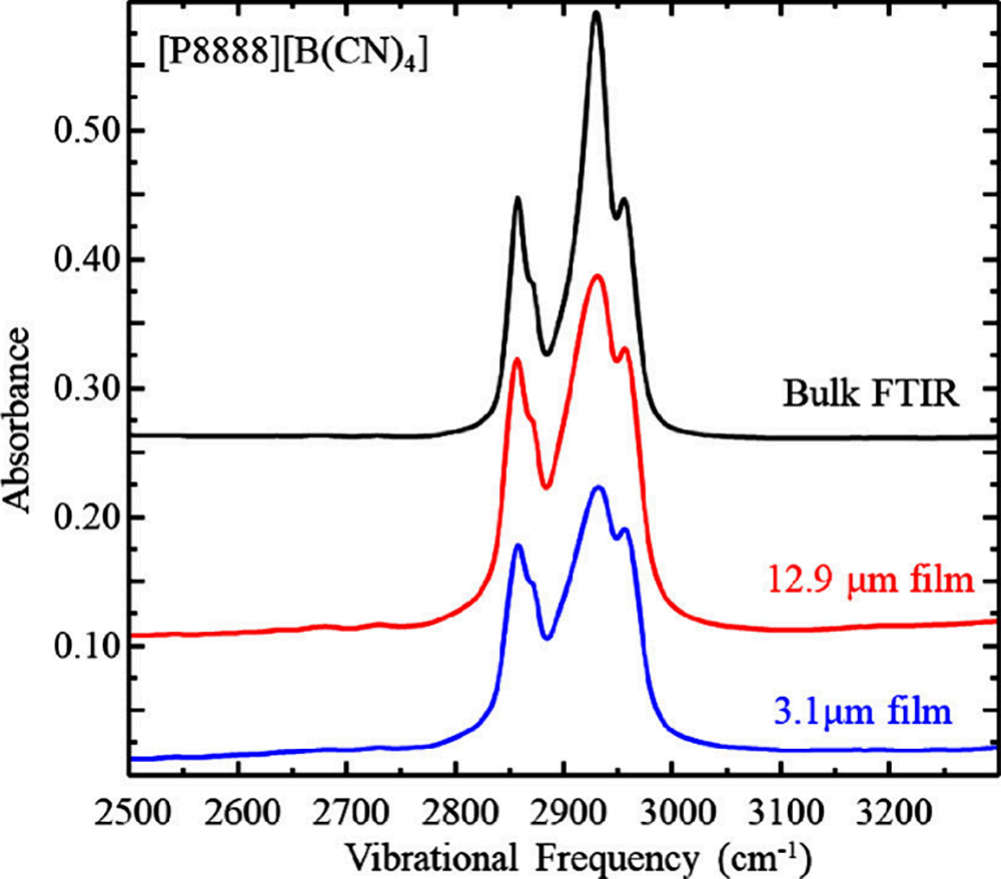
Infrared spectra of [P8888][B(CN)_4_] aliphatic region acquired by transmission through bulk fluid (black), IRRAS on films rotating at 59 μms^–1^ (red) and matured for 10 h (blue), respectively. Data are representative of *n* ≥ 3 trials. These spectra are vertically offset for clarity.

**Figure 4:**
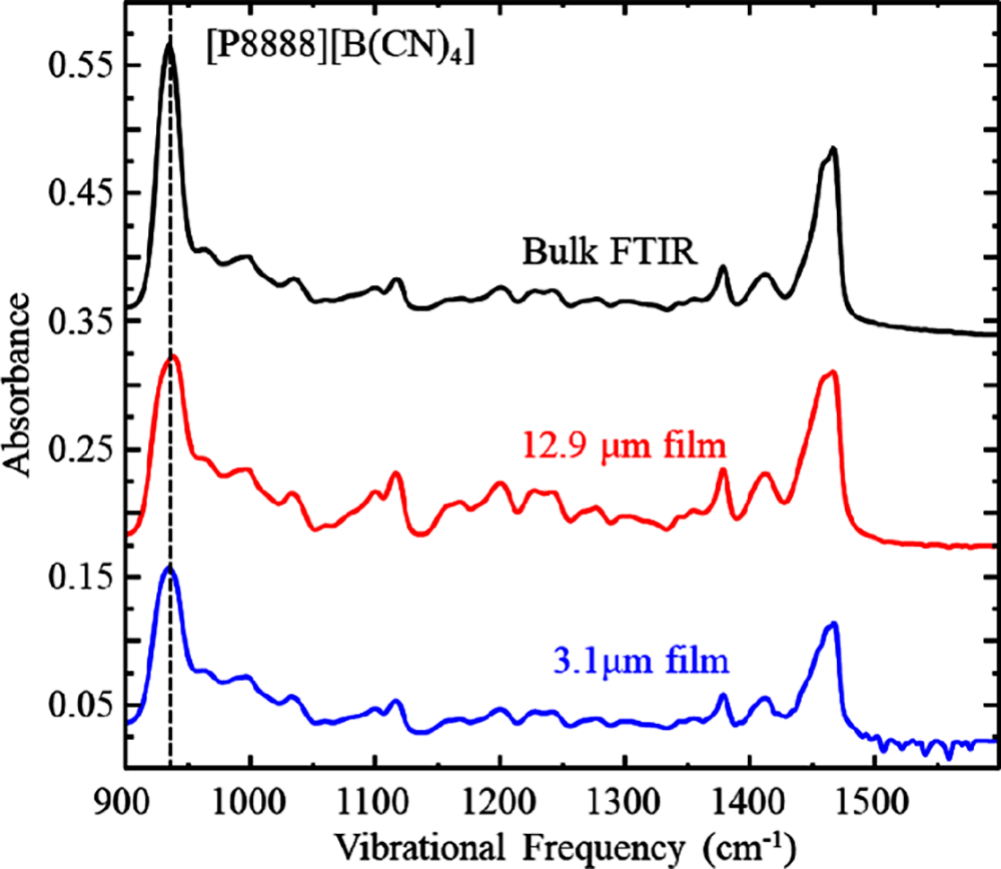
Infrared spectra of [P8888][B(CN)_4_] fingerprint region acquired by transmission through bulk fluid (black), IRRAS on films rotating at 59 μms^–1^ (red) and matured for 10 h (blue), respectively. Data are representative of *n* ≥ 3 trials. These spectra are vertically offset for clarity. Dashed vertical line marks the B–C stretch.

**Figure 5:**
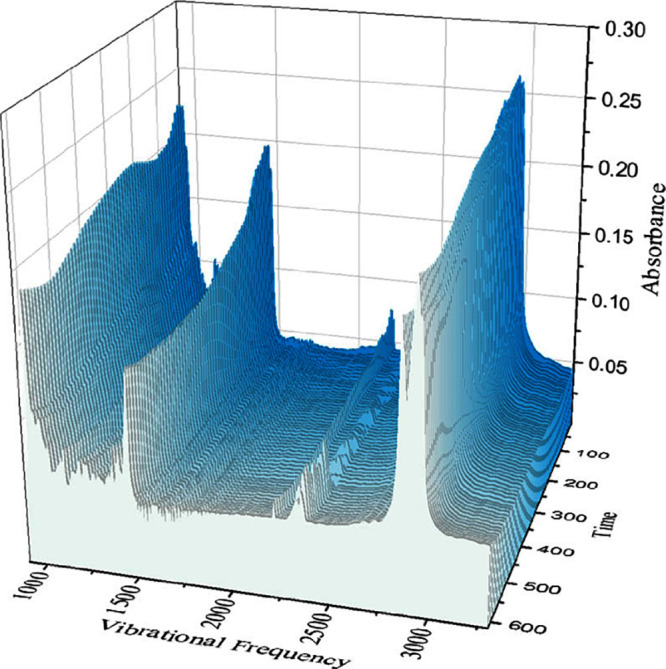
Waterfall plot for infrared spectra of [P8888][B(CN)_4_].

To track the maturation, or forming of ordered structures, within the IL films as has been reported previously,^23^ we monitored changes in the infrared spectroscopy data over time. Figures 3 and 4 show a series of infrared spectra acquired on [P8888][B(CN)_4_] showing the aliphatic, CH stretching region and the fingerprint region, respectively. The top spectrum corresponds to a transmission FTIR spectrum (black line), and the spectra below correspond to IRRAS spectra acquired while the film was rotating (red and blue lines). The high frequency range shows the aliphatic modes. The stretches of interest are at 2850 cm^–1^ (symmetric CH_2_), 2872 cm^–1^ (symmetric CH_3_), 2915 cm^–1^ (asymmetric CH_2_), 2956 cm^–1^ (asymmetric CH_3_), and the 2960 cm^–1^ (asymmetric CH_3_).^27^ The low frequency range shows the stretches associated with the fingerprint region. The two major stretches shown here are at 940 cm^–1^ (B–C stretch) and at 1450 cm^–1^ (C–N stretch). We include an infrared transmission spectrum of the same liquid for comparison, which highlights the similar vibrational modes observed for each liquid in these different environments. Figure 5 shows the evolution of these lower infrared energy absorption bands over time. Other than a decrease in absorbance as the film thins, which is to be expected as the effective path length is decreasing, there are no significant changes observed in the spectra. We contrast this data from our quasi-spherical ion liquid with the spectra acquired in prior work examining thin IL films with asymmetric cations and anions showing significant spectral changes characteristic of maturation, e.g. TFSI-based ILs showing four distinct vibrational mode shifts specifically in the 1000–1400 cm^–1^ range.^19,23^ Based on the lack of significant changes in the IR profiles with time, we suggest that the [P8888][B(CN)_4_] liquid film remains isotropic.

**Figure 6:**
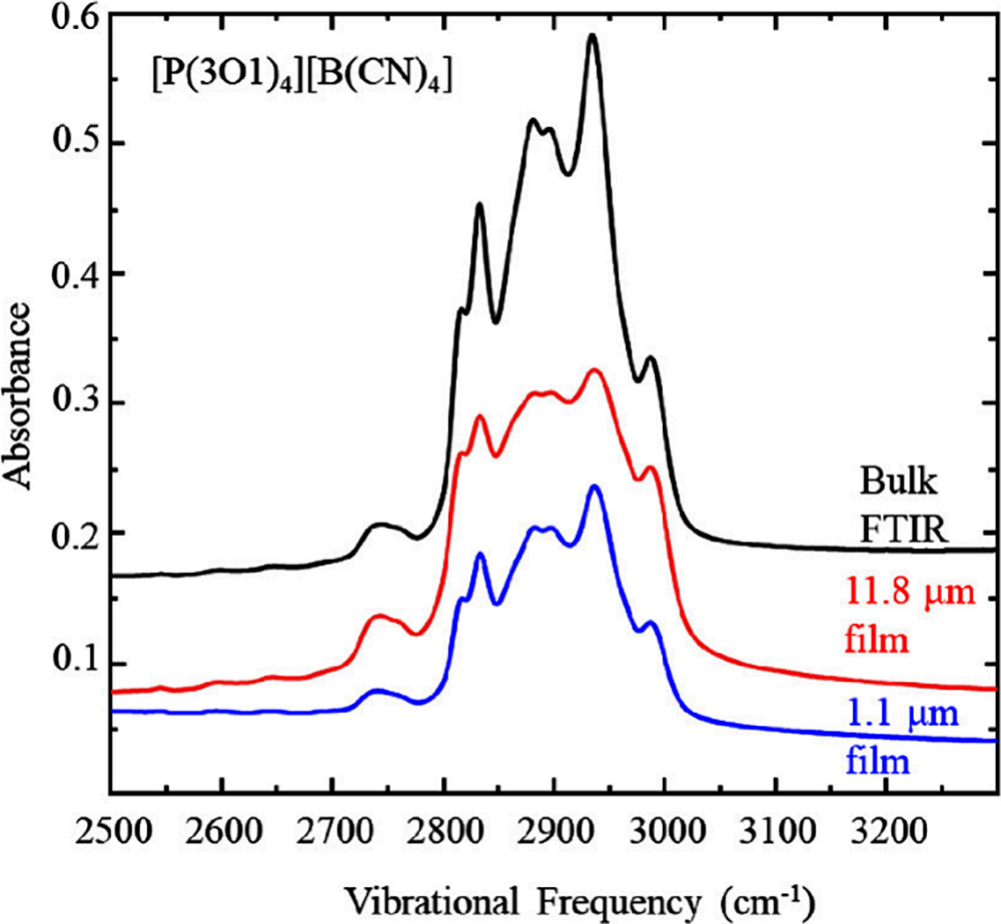
Infrared spectra of [P(3O1)_4_][B(CN)_4_] aliphatic region, acquired by transmission through bulk fluid (black), IRRAS on films rotating at 59 μm s^–1^ (red) and matured for 10 h (blue) respectively. Data are representative of *n* ≥ 3 trials. These spectra are vertically offset for clarity.

**Figure 7:**
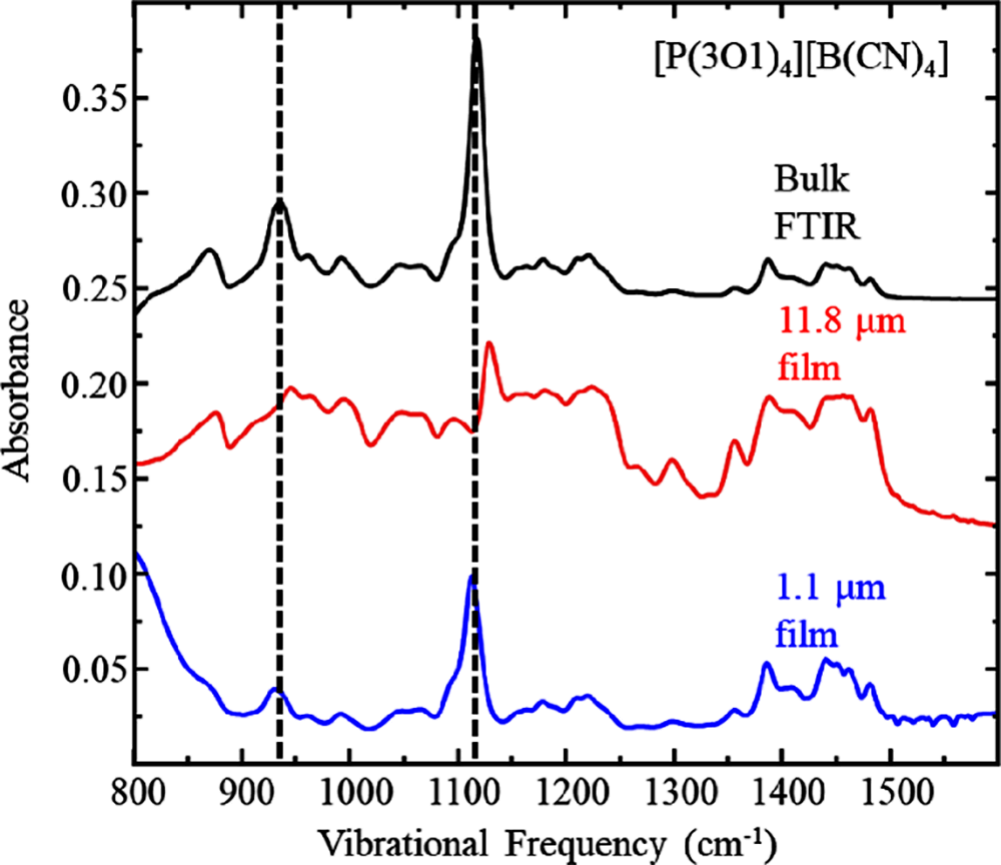
Infrared spectra of [P(3O1)_4_][B(CN)_4_] fingerprint region, acquired by transmission through bulk fluid (black), IRRAS on films rotating at 59 μms^–1^ (red, x3 scale) and matured for 10 h (blue, x10 scale) respectively. Data are representative of *n* ≥ 3 trials. These spectra are vertically offset for clarity.

**Figure 8:**
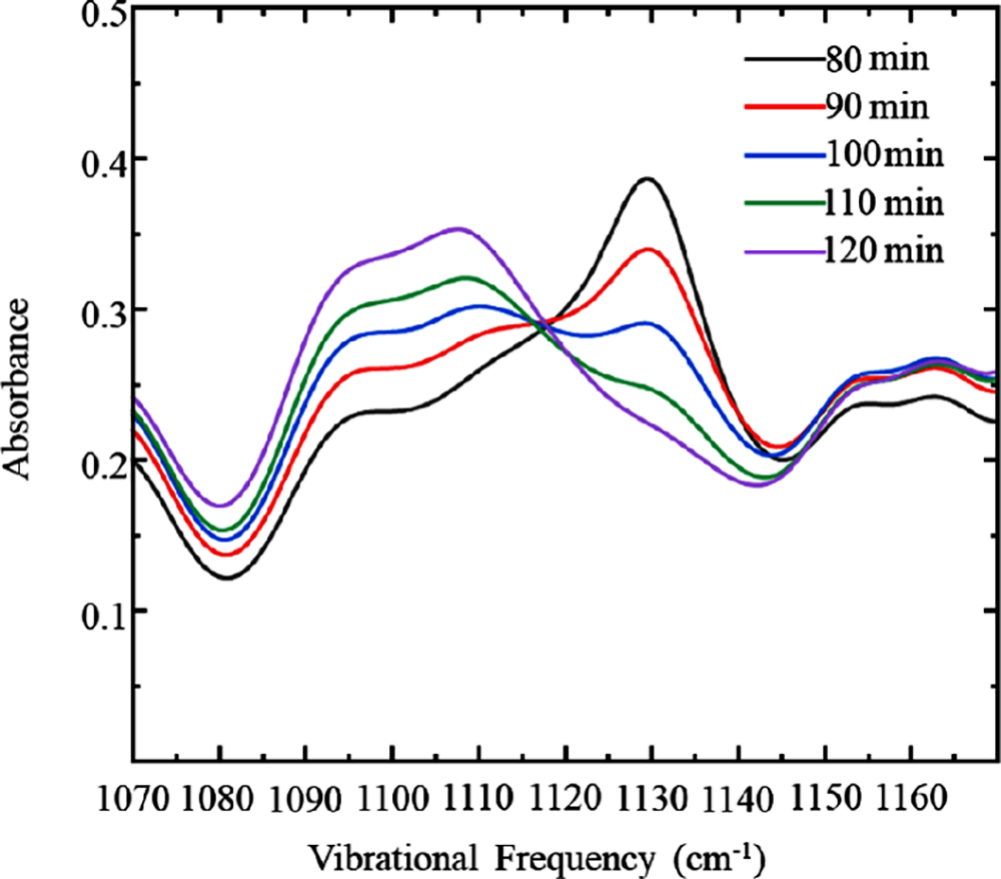
Infrared spectra of the [P(3O1)_4_][B(CN)_4_] ether mode acquired by IRRAS on the maturing film 80 to 120 min after ceasing rotation. Data are representative of *n* ≥ 3 trials.

**Figure 9:**
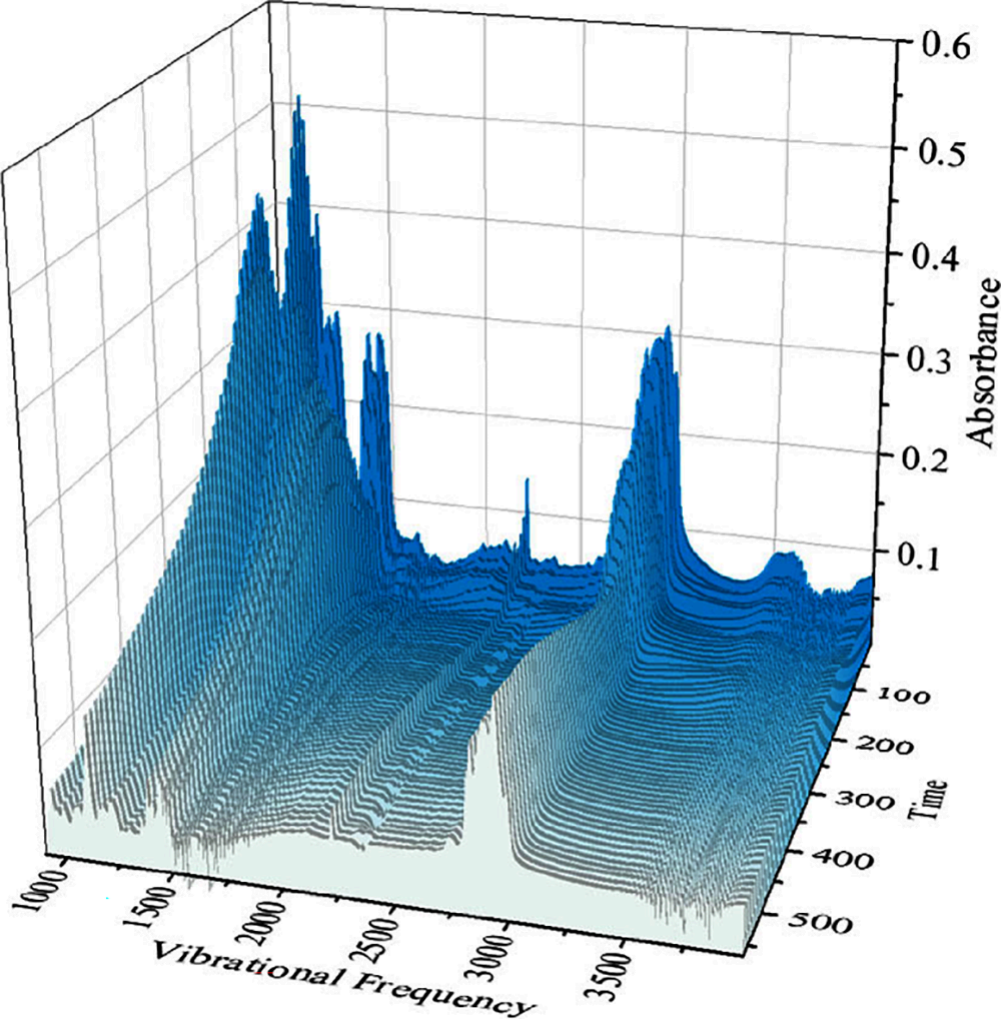
Waterfall plot for infrared spectra of [P(3O1)_4_][B(CN)_4_].

Figures 6 and 7 shows infrared data for the aliphatic and fingerprint regions of [P(3O1)_4_][B(CN)_4_] IL. The top (black) spectrum is a transmission FTIR spectrum and the spectra below correspond to IRRAS spectra acquired while the film is rotating (red and blue traces). Unlike the [P8888]^+^ IL, the rotating spectra have similar peak intensities in the aliphatic region for both the bulk and film phase suggesting that the chemical environments and molecular orientations within the films probed in these two cases remain similar over time. The low frequency fingerprint region shows two subtle but discernible changes in the IR profile. Specifically, the ether C–O stretch at ca. 1100 cm^–1^ (noted by a dashed vertical line in Figure 7) is observed in the bulk film, but changes frequency as time passes and the film thins in the rotating film spectra. Figure 8 depicts IR absorption spectra at selected time points of the [P(3O1)_4_][B(CN)_4_] film from 80 to 120 min, clearly showing that the C–O ether peak at 1128 cm^–1^ decreases commensurately with an increase in the 1107 cm^–1^ mode. We suggest this is due to a conformational change around the cation’s C–O–C group, where the ether tail begins to rotate toward the charged phosphonium head. This has been demonstrated previously in silico, showing ether tails can form “hairpin” structures^5^ which has in turn been shown to induce decreases in vibrational frequencies.^28^ Another minor change is seen in the IR profile for the B–C stretch which shifts ca. 10 wavenumbers to lower frequency over time (see Figure S1). The shift is completed within the first 150 min which coincides with the plateau in film thickness seen in Figure 2, suggesting the chemical environment of the film is stable while it continues to thin, slowly, for the duration of our measurements. Figure 9 shows a clearer view of this infrared spectra evolution over time, showing a decrease in absorbance as the film thins but no major frequency shifts or changes in full width at half maximum for the modes observed.

**Figure 10:**
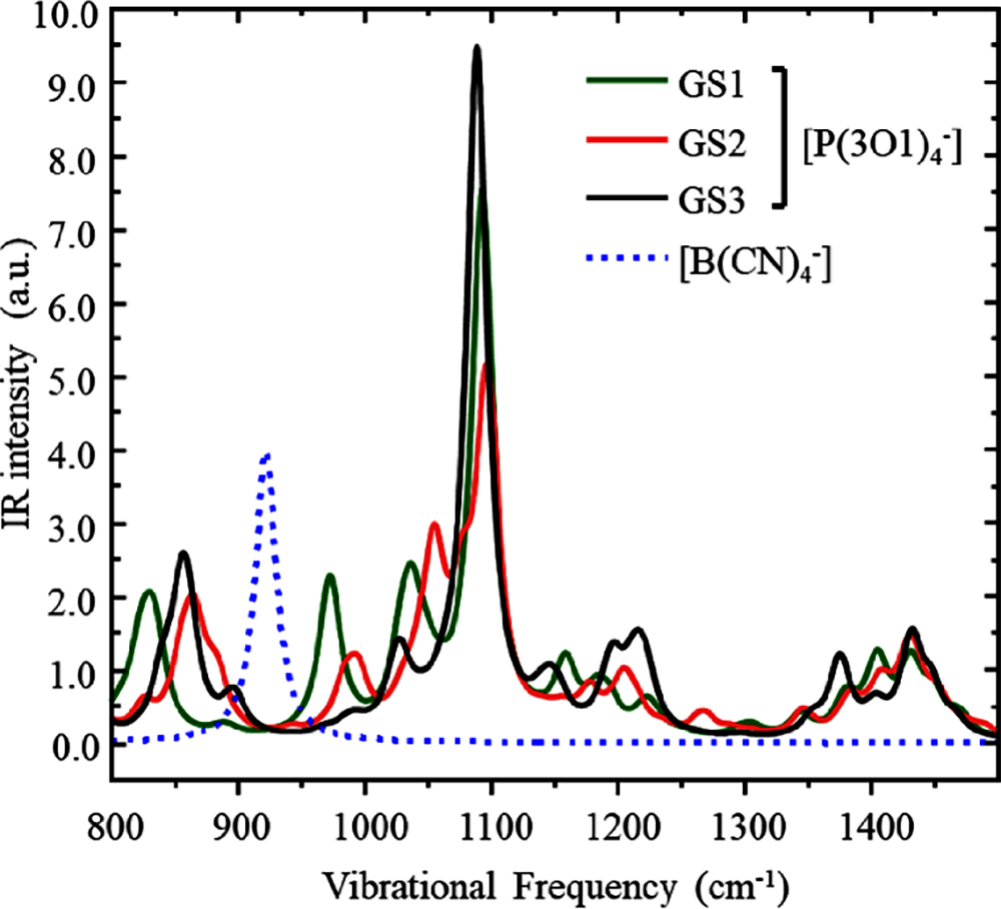
Ab initio simulated IR spectra for isolated ions in the gas phase. Three different conformers of the cation were considered.^13^ Peaks drawn with Lorentzian line broadening (fwhm 20 cm^–1^).

Figure 10 shows the three most likely vibrational modes from the theoretical gas phase spectra of [P(3O1)_4_] (green, red, and back), with [B(CN)_4_] shown in dashed blue. The signatures of the B–C stretch of the anion and the ether C–O stretch of the cation are clearly discernible. The experimentally observed broadening of the 1100 cm^–1^ feature, and the shift toward lower frequencies is consistent with a change from GS3 to either GS1 or GS2. In MD simulations of bulk [P(3O1)_4_][BF_4_], 34% of the phosphonium ion side chains were observed in GS2, and 55% in GS3.^21^ All other conformations including GS1 were much less prevalent. We thus suggest that the observed changes in the IR spectra are most likely due to an increase in the population of side chains in the GS2 conformer, at the expense of GS3.

Molecular dynamics simulations work has shown that ether tailed ILs exhibit dramatically different bulk structures from aliphatic tailed ILs.^29^ Replacing the aliphatic tail of an imidazolium-based IL with an ether tail caused the prepeak in simulated X-ray scattering data to disappear. This peak is associated with long-range interactions between charged head groups separated by apolar regions in ILs. Interestingly, this can yield faster dynamics in ether functionalized ILs, as the tail oxygen atoms compete with the charged anion, segregating cation from anion and weakening their coordination.^30^ The absence of this peak implies a less-ordered system, which is similar to what we see experimentally here e.g. the vibrational signature of the ether changing only slightly with respect to the significant changes observed previously for TFSI or triflate anion ionic liquids.^19^ The valuable comparison of the ether and aliphatic tails studied here suggests the aliphatic groups are mostly in the trans- orientation, which could be (as suggested by MD simulations) parallel to the IL charge network. Meanwhile, the ether tails curl back toward the cation core, reducing the overall cation symmetry. This reduced symmetry would explain the slight changes we observe in the infrared spectra over time: the ion orientations are no longer equivalent, which would lead to limited ordering (changes in the ether group IR energies).

A reorientation of the cation tails would also explain the slightly modified chemical environment of the anions (shifting B–C stretching frequencies). For the [PF_6_]^−^ anion, a similar shift to lower energies in the P–F stretch across increasing concentrations has been recorded before, suggesting some kind of stabilization.^31^ Ludwig et al. have also demonstrated that a reduced Coulombic coordination between cation and anion could lead to like-charge clustering in ILs,^32^ which has been shown with the [B(CN)_4_]^−^ anion specifically.^33^ The attractive dispersive and inductive interactions from the nitrile groups overcome anion–anion repulsion. If the ether tail reconfiguration would lead to a decrease in cation–anion coordination, it is quite possible the same anion–anion clustering is being observed here, explaining why the B–C stretch decreases energy in tandem with the ether mode shift.

## Conclusions

Films of completely symmetric ionic liquids should not undergo maturation due to their lack of a “preferred” (lowest energy) orientation. We have studied two ionic liquids, [P8888][B(CN)_4_] and [P(3O1)_4_][B(CN)_4_], that are nearly symmetrical, and the spectroscopic measurements indicate suppressed ordering when compared to their more asymmetric counterparts, e.g., the series of alkyl-imidazolium triflates. Ultimately, we report that the [P8888][B(CN)_4_] film does not show significant signs of maturation which supported our hypothesis; however, the [P(3O1)_4_][B(CN)_4_] film did show subtle signs of maturation/orientation. We attributed these slight changes to deformation of the ether tails around the phosphorus atom core: as the ether tails curl back to interact with the charged phosphorus, they induce a reduced symmetry of this cation.

## Supporting Information Available

Center frequency of the vibrational mode assigned to the B–C stretch acquired on [P(3O1)_4_][B(CN)_4_] and [P8888][B(CN)_4_] (PDF)
