# Supplementary material for: Increasing Cation Ion Symmetry Reduces Ionic Liquid Ordering in Thin Films
Source: J Phys Chem B. 2024 Nov 5;128(45):11251–7. doi: 10.1021/acs.jpcb.4c04413 (PMC11571225; doi:10.1021/acs.jpcb.4c04413)
Supplement: Supplementary file 1 — jp4c04413_si_001.pdf [file jp4c04413_si_001.pdf]

Supporting Information for

Increasing cation ion symmetry reduces ionic liquid ordering in thin films

Author information:

Van Den Top, Michael Blake[1] ; mvandentop@uiowa.edu 0009-0007-1631-4759; University of Iowa

Horvath, Andrew[1] ; Andrew-horvath@uiowa.edu 0000-0001-9067-3206; University of Iowa

Koutsoukos, Spyridon[2] ; s.koutsoukos18@imperial.ac.uk; 0000-0003-2546-5317; Imperial College London

Philippi, Frederik[2] ; f.philippi18@imperial.ac.uk 0000-0002-0127-8923; Imperial College London

Daniel Rauber ; daniel.rauber@uni-saarland.de 0000-0003-0488-4344; Saarland University

Welton, Tom*[2] ; t.welton@imperial.ac.uk 0000-0002-1750-1683; Imperial College London

Shaw, Scott K.*[1] ; scott-k-shaw@uiowa.edu 0000-0003-3767-3236; University of Iowa

Institutions:

1. Department of Chemistry, University of Iowa, Iowa City, Iowa 52242, United States
2. Department of Chemistry, Imperial College London SW7 2AZ, UK
3. Department of Chemistry, Saarland University, Campus B 2.2, 66123 Saarbrücken, Germany

Contents:

Figure S1: Vibrational frequency of the B-C mode vs time Page 2

Figure S1:

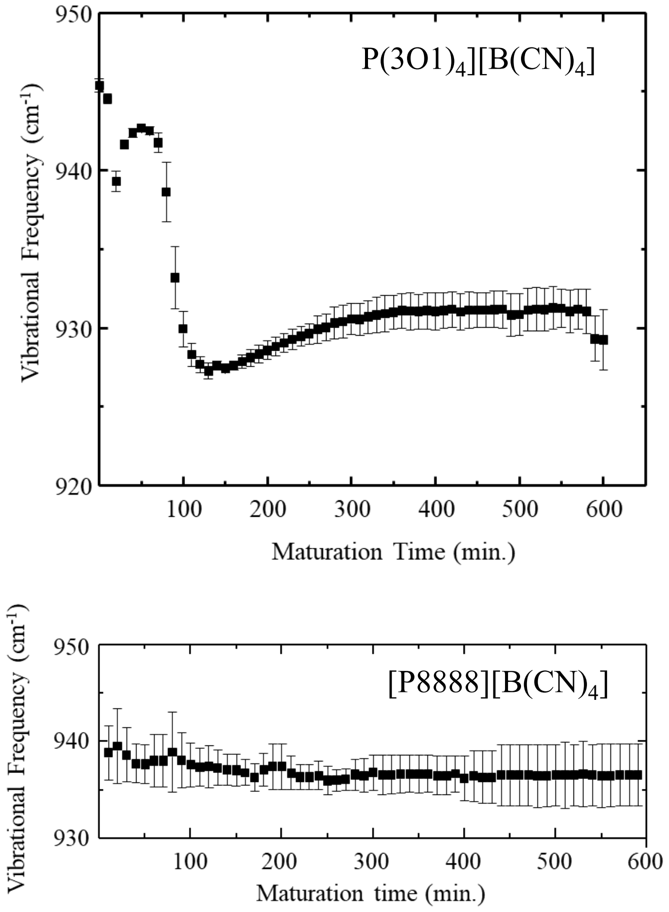

TOP: The center frequency of the vibrational mode assigned to the B-C stretch acquired on $[P(3O1)_4][B(CN)_4]$ as a function of time after substrate rotation is ceased. Data points (black squares) are representative of $n \geq 3$ trials. Error bars represent standard deviation in the measurement.

BOTTOM: The center frequency of the vibrational mode assigned to the B-C stretch acquired on $[P8888][B(CN)_4]$ as a function of time after substrate rotation is ceased. Data points (black squares) are representative of $n \geq 3$ trials. Error bars represent standard deviation in the measurement.
